# Effectiveness of a Community-Based Social Innovation for *Opisthorchis viverrini* and Cholangiocarcinoma Prevention in High-Risk Areas of Thailand

**DOI:** 10.31557/APJCP.2026.27.1.353

**Published:** 2026-01-22

**Authors:** Comsun Thongchai, Ratchanee Joomjee, Orathai Srithongtham, Getsara Sansiritawisuk, Nopparat Songserm

**Affiliations:** 1 *Department of Public Health, Faculty of Public Health, Ubon Ratchathani Rajabhat University, Ubon Ratchathani, Thailand.*; 2 *Department of Occupational Health and Safety, Faculty of Public Health, Ubon Ratchathani Rajabhat University, Ubon Ratchathani, Thailand.*; 3 *The Office of Disease Prevention and Control 10, Ubon Ratchathani, Thailand.*; 4 *Department of Health Sciences, Faculty of Public Health, Ubon Ratchathani Rajabhat University, Ubon Ratchathani, Thailand.*

**Keywords:** Cholangiocarcinoma, Health behavior, Opisthorchis viverrine, Social innovation

## Abstract

**Objectives:**

This study aimed to develop and evaluate a community-based social innovation to prevent *Opisthorchis viverrini* (OV) infection and cholangiocarcinoma (CCA), which are critical public health challenges in northeastern Thailand.

**Methods:**

A research and development (R&D) approach was implemented across five provinces in Thailand’s 10th Health Region. The intervention, “3 Health for a CCA-Free Society,” focused on three domains: Health Behaviors, Health Hygiene, and Environmental Health. A manual-based innovation was co-developed through community engagement and expert collaboration. Using a quasi-experimental pre-post design, its effectiveness was evaluated in a prototype area that was randomly selected from the five provinces (n = 56). Structured questionnaires assessed participants’ knowledge, health beliefs, and preventive behaviors. Paired t-tests and Wilcoxon signed-rank tests were applied to evaluate changes. Satisfaction was assessed using the Context, Input, Process, and Product (CIPP) model.

**Results:**

Statistically significant improvements were found in knowledge (mean increase = 0.47, p < 0.001), health beliefs (mean increase = 3.92, p < 0.001), and preventive behaviors (mean increase = 3.07, p < 0.001). Satisfaction scores were high across all CIPP domains (mean = 4.88 ± 0.26). The manual was rated clear, relevant, and culturally appropriate, indicating strong potential for broader application.

**Conclusion:**

This community-based social innovation significantly improved OV and CCA prevention outcomes in a high-risk area. The approach is feasible, scalable, and aligned with national strategies for participatory disease prevention. Further studies should explore its long-term impact and the potential for digital adaptation for broader dissemination.

## Introduction

Cholangiocarcinoma (CCA) remains a critical public health concern in Southeast Asia, especially in Thailand, where *Opisthorchis viverrini* (OV) infection is a significant risk factor for bile duct cancer. Despite decades of national control efforts including public campaigns, praziquantel deworming, and health education, the burden of OV infection persists in endemic areas, contributing to high rates of CCA [1, 2]. A spatial analysis of hepatobiliary abnormalities in high-risk populations confirmed that northeastern Thailand, particularly provinces such as Ubon Ratchathani, Sisaket, Yasothon, Mukdahan, and Amnat Charoen, represents the area with the highest burden of OV infection and CCA [3].

To address this ongoing challenge, Thailand’s Ministry of Public Health implemented a 20-year national strategic plan (2018–2037) to strengthen disease prevention and control through decentralized, community-based interventions [4]. Within this context, social innovation has emerged as a key mechanism for public health reform. Social innovations are novel, collaborative approaches that address complex societal issues through community engagement, multisectoral cooperation, and locally adapted solutions [5, 6]. These approaches have proven effective in transforming health behaviors, improving access to health services, and promoting health equity—particularly in under-resourced settings, as highlighted by a scoping review demonstrating the growing application of social innovation in healthcare to enhance equity and access in low- and middle-income countries [7].

Building on Thailand’s 20-year National Strategic Plan (2018–2037) and the social innovation in health framework, which emphasizes decentralized, community-based interventions, community engagement, and multisectoral cooperation, this study represents the second phase of a research initiative aimed at preventing and controlling OV and CCA in the highest-risk areas of Thailand. Phase 1 used a qualitative approach to examine stakeholder experiences and identify promising community-based strategies and grassroots innovations in five endemic provinces [8]. These insights laid the groundwork for Phase 2, which sought to transform successful elements into a manual-based social innovation. A key outcome was the development of a community manual titled “3 Health for a CCA-Free Society,” designed as a practical and culturally grounded manual-based social innovation tool. Whereas the first phase emphasized problem identification and conceptual mapping, this second phase focused on developing, implementing, and evaluating an adaptable manual-based social innovation for similar high-risk communities. The “3 Health” framework comprises three core domains: Health Behaviors, Health Hygiene, and Environmental Health, each reflecting locally relevant risk factors associated with OV and CCA.

Recent evidence underscores the potential of manual-based, community-led social innovations in public health. A mixed-methods systematic review reported that participatory strategies guided by context-specific manuals significantly enhanced disease prevention behaviors and strengthened community resilience in low- and middle-income countries [9]. Similarly, a case study in Cambodia showed that co-developed manuals for dengue control increased adaptive capacity and promoted sustained behavior change through active community engagement [10]. Drawing on these precedents, the present study evaluated the effectiveness of the 3 Health for a CCA-Free Society manual in improving participants’ knowledge, health beliefs, and preventive behaviors related to OV and CCA. The findings aim to inform evidence-based policy development and offer a scalable model for participatory disease prevention in resource-limited or high-burden settings. Specifically, the study sought to assess changes in knowledge, health beliefs, and preventive practices following exposure to manual-based social innovation.

## Materials and Methods

### Study design and setting

This study applied a research and development (R&D) design to develop and assess the effectiveness of a manual-based social innovation for the prevention and control of OV and CCA. The study was conducted from July 2022 to March 2023 in Thailand’s 10th Health Region, comprising five provinces. The R&D design was selected to facilitate the systematic creation, pilot testing, and effectiveness evaluation of the innovation in community-based settings. The process emphasized community engagement, contextual relevance, and iterative refinement, employing qualitative and quantitative methods. The final product, a community manual titled “3 Health for a CCA-Free Society” was developed to address locally specific risk factors across three domains: Health Behaviors, Health Hygiene, and Environmental Health. The study incorporated formative and summative phases, engaging local stakeholders throughout the development process and utilizing a quasi-experimental pre-post design to evaluate changes in knowledge, health beliefs, and preventive behaviors related to OV and CCA.

### Study participants

This study involved participants at different stages of the R&D process. In Phase R1, local stakeholders from five high-risk provinces in northeastern Thailand were engaged in data collection to identify existing social innovations for OV and CCA prevention. In Phase D1, selected preventive medicine and public health experts collaborated with the research team to design the innovation. In Phase R2, a pilot group of 25 participants from two communities in Yang Yai Subdistrict, Nam Yuen District, Ubon Ratchathani Province, participated in testing the initial version of the innovation. In Phase D2, the refined version was implemented with 56 participants in Nong Bo Subdistrict, Mueang District, Ubon Ratchathani Province, a prototype community randomly selected from the five provinces to assess the innovation’s effectiveness and readiness for broader application.

The sample size for the study group was determined using G*Power 3.1.9.7, based on power analysis for a quasi-experimental one-group design [11]. With an effect size of 0.90, alpha = 0.05, and power = 0.90, the minimum required sample size was 46. A 20% buffer was added to allow for potential attrition, yielding a final sample size of 56 participants.

Participants were eligible if they (1) were at least 20 years old, (2) resided in the study community, and (3) were free from OV infection or CCA at the time of recruitment. Those with severe illness, cognitive impairment, or unwillingness to participate were excluded.

### Research instrument development

This study employed three primary research tools: (1) a manual-based social innovation for the prevention and control of OV and CCA; (2) a structured questionnaire designed to assess participants’ knowledge, health beliefs, and preventive behaviors related to OV and CCA; and (3) a satisfaction evaluation form to measure participants’ opinions toward the innovation manual.

The social innovation “3 Health for a CCA-Free Society” was developed during the first and second phases of the research and was grounded in empirical data from five high-risk provinces in northeastern Thailand. The manual consisted of three modules addressing Health Behaviors (e.g., avoiding raw fish consumption, alcohol abstinence during merit-making events, regular screening), Health Hygiene (e.g., use of sanitary toilets, proper fecal disposal), and Environmental Health (e.g., elimination of snail habitats, reduction of water contamination). To ensure community relevance and practical use, the content was delivered through participatory learning methods such as group discussions, community meetings, role-playing, and visual aids. Printed manuals were distributed to support individual and household-level applications.

### Validity and reliability testing

The questionnaire and satisfaction form underwent content validation and reliability testing to ensure instrument quality. Five experts in OV and CCA prevention and control assessed content validity using a 4-point relevance scale (1 = not relevant, 4 = highly relevant). For each item, the Item-Level Content Validity Index (I-CVI) was calculated as the proportion of experts rating the item as 3 or 4. The Scale-Level Content Validity Index (S-CVI) was then computed as the average of the I-CVI values across all items, yielding an S-CVI of 0.81.

Reliability testing was conducted with 30 community members in Ummao Subdistrict, Roi Et Province. For Likert-scale items (health beliefs, preventive behaviors, and satisfaction form), internal consistency was assessed using Cronbach’s alpha. The resulting coefficients were 0.83 for the health beliefs section, 0.79 for preventive behaviors, and 0.91 for the satisfaction form, indicating acceptable to excellent reliability. For the knowledge section, which included binary (yes/no) items, the Kuder-Richardson formula (KR-20) was applied, yielding a coefficient of 0.75.

The questionnaire was structured into four parts: Part 1: Demographic information gender, age, marital status, education, occupation, income, and underlying health conditions. Part 2: Knowledge of OV and CCA prevention (10 items), scored Yes = 1, No = 0, with score ranges interpreted as high (≥80%), moderate (60–79%), and low (<60%) based on Bloom’s taxonomy [12]. Part 3: Health beliefs (20 items) based on the Health Belief Model, rated on a 3-point scale: Agree (3), Unsure (2), Disagree (1). Scoring interpretation: high (2.34–3.00), moderate (1.67–2.33), low (1.00–1.66) [13]. Part 4: Preventive behaviors (10 items), also using a 3-point scale: Always (3), Sometimes (2), Never (1), with similar scoring interpretation.

The satisfaction evaluation form consisted of 12 items rated on a 5-point Likert scale, ranging from Strongly Agree (5) to Strongly Disagree (1). The interpretation of mean scores was classified as follows: highest satisfaction (4.20–5.00), high (3.40–4.19), moderate (2.60–3.39), low (1.80–2.59), and lowest (1.00–1.79) [13]. This instrument assessed participants’ overall impressions of the manual regarding content, clarity, relevance, and usefulness.

### Study procedure

**Figure 1:**
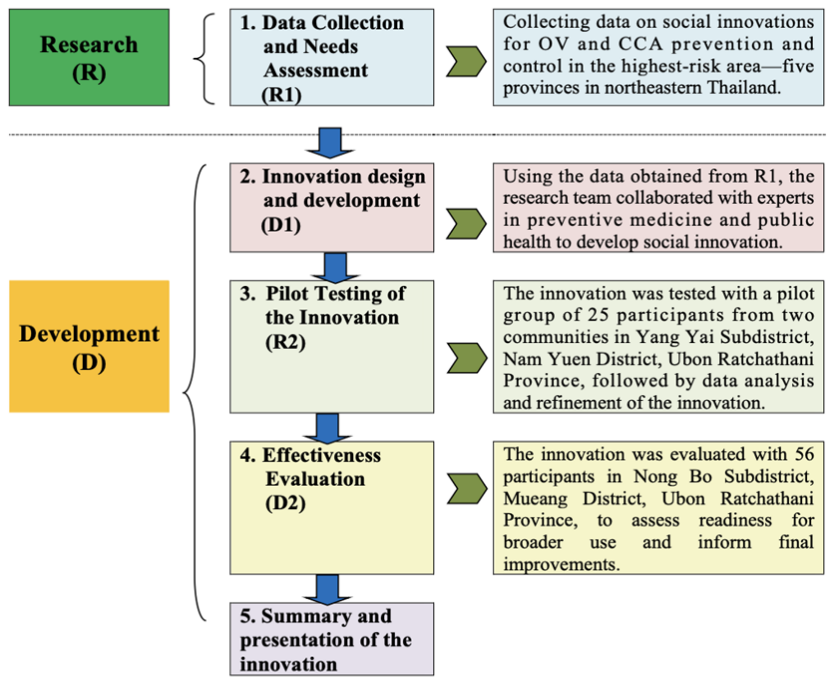
Research and Development (R&D) Process for Developing a Manual-based Social Innovation to Prevent *Opisthorchis viverrini* and Cholangiocarcinoma. The five-phase process included needs assessment (R1), innovation design (D1), pilot testing (R2), effectiveness evaluation (D2), and finalization for community use.

This study employed an R&D design and was conducted between July 2022 and March 2023 in Thailand’s 10th Health Region, comprising five northeastern provinces identified as high-risk areas for OV infection and CCA. The study followed five key steps based on the R&D process, as illustrated in Figure 1.

### Data collection and needs assessment (R1)

A situational analysis was conducted in five endemic provinces (Ubon Ratchathani, Sisaket, Yasothon, Mukdahan, and Amnat Charoen) to identify existing community-based innovations for OV and CCA prevention and control. This phase involved field observations, in-depth interviews, and focus group discussions with local stakeholders, including community leaders, health volunteers, and public health officers.

### Innovation design and development (D1)

Findings from Phase 1 were used to develop a manual-based social innovation titled “3 Health for a CCA-Free Society.” This development was conducted through collaboration between the research team and experts in preventive medicine and public health. The manual integrated three thematic modules Health Behaviors, Health Hygiene, and Environmental Health. Participatory learning methods were applied to ensure the content was culturally appropriate and community-relevant.

### Pilot testing of the innovation (R2)

The innovation was piloted with 25 participants from two communities in Yang Yai Subdistrict, Nam Yuen District, Ubon Ratchathani Province. Participants engaged in the program through community workshops using the manual. Feedback and preliminary outcomes were collected to revise and improve the innovation’s structure, clarity, and delivery approach.

### Effectiveness evaluation (D2)

The refined innovation was implemented with 56 participants in Nong Bo Subdistrict, Mueang District, Ubon Ratchathani Province. A structured pre- and post-assessment was conducted to evaluate changes in participants’ knowledge, health beliefs, and preventive behaviors related to OV and CCA. Satisfaction with the manual and delivery format was also assessed to determine readiness for broader application.

### Summary and presentation of the innovation

Based on findings from the evaluation phase, final adjustments were made to the manual. The completed innovation was documented for future use and potential scale-up in other high-risk communities. The final product was designed to be accessible, sustainable, and adaptable to various community settings.

### Statistical analysis

All data were analyzed using the Statistical Package for the Social Sciences (SPSS) version 29.0 (IBM Corp., Armonk, NY, USA). Descriptive statistics (frequency, percentage, mean, and standard deviation) were used to summarize demographic characteristics and participants’ responses regarding knowledge, health beliefs, preventive behaviors, and satisfaction with the innovation manual. Normality was assessed using the Shapiro–Wilk test. Paired sample t-tests were applied to compare pre- and post-intervention mean scores for normally distributed variables, whereas the Wilcoxon signed-rank test was used for non-normally distributed variables. For the pilot group (n = 25), the median with interquartile range (IQR) was reported, and box plots were generated to visualize score distributions and potential outliers. Effect sizes and 95% confidence intervals were reported where appropriate. Statistical significance was set at p < 0.05.

## Results

The “3 Health for a CCA-Free Society” social innovation was developed and evaluated through a structured R&D process comprising five sequential phases (R1–D2), as illustrated in Figure 1.

### Data collection and needs assessment (R1)

In-depth fieldwork was conducted across five provinces in Thailand’s 10th Health Region to identify persistent OV and CCA risk factors. Focus group discussions with community stakeholders revealed continued consumption of raw freshwater fish, limited awareness of OV transmission, inadequate sanitation, and environmental conditions conducive to parasite survival. These findings informed the design of a culturally tailored and behaviorally relevant innovation framework.

### Innovation design and development (D1)

Based on R1 findings, the research team collaborated with public health experts and local representatives to co-develop a manual-based innovation focused on three domains: Health Behaviors, Health Hygiene, and Environmental Health. The manual incorporated participatory learning strategies and underwent expert review to ensure contextual relevance and practical feasibility.

### Pilot testing of the innovation (R2)

**Figure 2:**
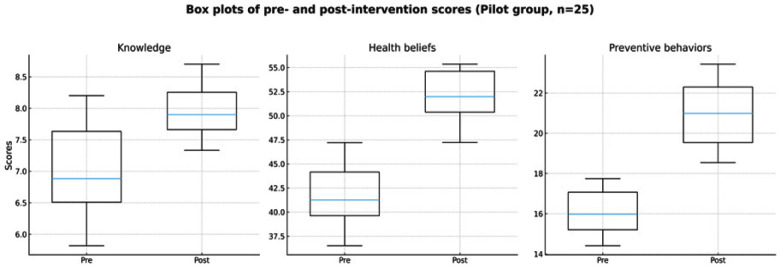
Box Plots Showing Pre- and Post-Intervention Distributions of Scores for Knowledge, Health Beliefs, and Preventive Behaviors among the Pilot Group (n = 25). Median scores and interquartile range improved significantly across all domains after the intervention (Wilcoxon signed-rank test, p < 0.001).

The innovation was piloted with 25 participants in the Yang Yai Subdistrict. The Wilcoxon signed-rank test revealed statistically significant improvements across all domains. Median knowledge scores increased from 7.0 (IQR = 2.50) to 8.0 (IQR = 1.50) (Z = –4.064, p < 0.001), health beliefs from 42.0 (11.00) to 51.0 (9.00) (Z = –4.109, p < 0.001), and preventive behaviors from 16.0 (3.50) to 21.0 (5.00) (Z = –3.529, p < 0.001), as shown in Table 1. Box plots illustrated upward shifts in score distributions with reduced variability following the intervention (Figure 2). Participant feedback indicated that the manual was clear, relevant, and applicable, with recommendations to enhance visual design and adjust content pacing.

### Effectiveness evaluation (D2)

Following refinement, the final version of the innovation was implemented with 56 participants in Nong Bo Subdistrict using a quasi-experimental design. Statistically significant post-intervention improvements were observed in knowledge (mean increase: 0.47, p < 0.001), health beliefs (mean increase: 3.92, p < 0.001), and prevention behaviors (mean increase: 3.07, p < 0.001), confirming the intervention’s effectiveness (Table 2). While the magnitude of change was slightly lower than in the pilot phase, the direction of improvement was consistent across all domains.

### Satisfaction evaluation based on the CIPP Model

Participant satisfaction with the innovation manual was assessed using the CIPP Model framework Context, Input, Process, and Product as shown in Table 3. All 12 evaluation items received ratings in the “highest” category, with an overall mean score of 4.88 (SD = 0.26). Notably, full scores (5.00) were recorded in key areas such as content relevance, accuracy, and knowledge transfer capability. These results underscore the manual’s clarity, cultural appropriateness, and strong potential for adoption in OV-endemic community settings.

These findings suggest that manual-based social innovation can be a scalable and sustainable OV and CCA prevention approach, particularly in resource-limited and culturally diverse communities.

## Discussion

This study demonstrated that the “3 Health for a CCA-Free Society” social innovation comprising Health Behaviors, Health Hygiene, and Environmental Health effectively enhanced participants’ knowledge, health beliefs, and prevention behaviors regarding OV and CCA in a high-risk area of Thailand. These findings highlight the utility of manual-based social innovation in driving behavior change toward OV and CCA prevention.

The results support previous studies emphasizing the importance of locally adapted educational tools and participatory approaches. For instance, a health literacy promotion program significantly improved participants’ knowledge, access to health services, and decision-making capacity [14]. Similar outcomes were reported through a web-based application to promote awareness and behavioral change related to OV infection [15]. Community mobilization and structured health education have also been highlighted as key strategies for CCA prevention [16, 17]. This study reinforces those findings by demonstrating that a culturally grounded and empirically developed manual delivered through interactive community activities can effectively increase disease-related knowledge and influence beliefs and behaviors. The improvement in knowledge and practices related to OV and CCA may also reflect the strength of the participatory learning process embedded in the program, which emphasizes dialogue, shared experience, and locally relevant examples.

The innovation manual received high satisfaction scores across all components evaluated under the CIPP Model Context, Input, Process, and Product. Participants rated the content as accurate, relevant, and clearly presented while appreciating the facilitators’ ability to deliver the material effectively. These satisfaction results suggest strong community acceptance of the intervention and indicate the manual’s feasibility in other OV-endemic communities. This aligns with systematic review findings showing that educational programs assessed via the CIPP model frequently receive high satisfaction ratings, particularly when they involve experienced facilitators and well-structured, contextually appropriate content [18]. Such evidence further validates manual-based innovations’ credibility and adaptability across diverse public health contexts.

Notably, the structured R&D process adopted in this study ensured iterative innovation refinement based on real-time stakeholder feedback, expert validation, and pilot testing. This approach aligns with the social R&D framework [6] and the innovation development model [19], emphasizing co-creation, contextual sensitivity, and knowledge translation into practice. The findings also align with international evidence on the role of social innovation in public health. One study highlighted the benefits of community-driven manuals for dengue control in Cambodia [10]. At the same time, another found that manual-guided, participatory strategies improved disease prevention behaviors and strengthened community resilience in low- and middle-income countries [9]. These parallels reinforce the relevance of the current model beyond Thailand’s context. Importantly, the observed improvements in preventive behaviors such as avoiding raw fish consumption and participating in health screenings are critical steps toward interrupting the transmission cycle of OV and reducing long-term CCA risk. Such behavior changes, when sustained, can have meaningful public health impacts in endemic settings where conventional top-down strategies may be less effective.

### Limitations

This study has several limitations. First, the one-group quasi-experimental design without a control group limits causal inference; future studies should adopt a control group or randomized controlled trial (RCT). Second, the findings reflect only short-term outcomes, and the sustainability of behavioral change remains unclear; long-term follow-up is needed to examine factors influencing maintenance of preventive behaviors. Third, reliance on self-reported questionnaires may have introduced recall or social desirability bias. Fourth, the small sample size from a single province limits generalizability to other OV-endemic communities in Thailand or neighboring countries, and findings may apply only to communities with similar cultural and geographic characteristics. Future research should address these limitations through controlled trials, extended follow-up, and larger, more diverse populations.

In conclusion, the “3 Health for a CCA-Free Society” social innovation demonstrated effectiveness in improving participants’ knowledge, health beliefs, and preventive behaviors related to OV and CCA in a high-risk community in Thailand. The manual-based approach grounded in a community context and developed through a structured R&D process proved feasible and well-accepted. The high satisfaction ratings across all components of the CIPP Model reinforce its relevance and applicability for use in OV-endemic areas. This intervention represents a promising model for culturally appropriate, community-driven health education that can be adapted for broader public health applications.

### Recommendations

Based on the study’s findings, several recommendations are proposed: 1) Manual-based innovation should be considered for broader implementation in other OV-endemic communities, particularly within Thailand’s national OV and CCA prevention strategy. 2) Future research should include extended follow-up periods to assess the sustainability of behavior change and long-term health outcomes. 3) Incorporating control groups in future studies will strengthen the evidence base and provide clearer insights into the intervention’s effectiveness. 4) To enhance accessibility and reach, the innovation could be adapted into digital formats, including mobile applications or e-learning platforms, especially for remote or younger populations. 5) The findings should be shared with regional health authorities and policymakers to support the integration of social innovation approaches into existing health promotion frameworks.

**Table 1:** Comparison of Pre- and Post-Intervention Median Scores for Knowledge, Health Beliefs, and Preventive Behaviors Related to *Opisthorchis viverrini* and Cholangiocarcinoma among Pilot Group (n = 25).

Factors	Before Median (IQR)	After Median (IQR)	Mean Rank	Sum of Ranks	Wilcoxon signed-rank test (Z)	p-value
Pilot group (n = 25)						
l Knowledge of OV and CCA prevention and control	7 (2.50 )	8 (1.50 )	10.5	210	-4.064*	<0.001
l Health beliefs in disease prevention and control	42 (11.00)	51 (9.00)	11.5	253	-4.109*	<0.001
l OV and CCA prevention and control behaviors	16 (3.50 )	21 (5.00 )	8.5	136	-3.529*	<0.001

CCA, Cholangiocarcinoma; IQR, Interquartile range; OV, *Opisthorchis viverrini;* *Based on negative ranks

**Table 2:** Comparison of Pre- and Post-Intervention Mean Scores for Knowledge, Health Beliefs, and Preventive Behaviors Related to* Opisthorchis viverrini *and Cholangiocarcinoma among Study Groups (n = 56).

Factors	Before Mean ± SD	After Mean ± SD	Mean Difference	95% CI	p-value
Study group (n = 56)					
Knowledge of OV and CCA prevention and control	6.51 ± 1.53	6.88 ± 1.79	0.47	0.31 - 0.62	<0.001
Health beliefs in disease prevention and control	41.33 ± 8.44	45.25 ± 7.88	3.92	1.18 - 6.06	<0.001
OV and CCA prevention and control behaviors	17.85 ± 3.37	20.92 ± 2.89	3.07	2.12 - 4.20	<0.001

CCA, Cholangiocarcinoma; OV, *Opisthorchis viverrini;* SD, Standard deviation; 95% CI, 95% Confidence Interval

**Table 3:** Participant Satisfaction Scores and Interpretation based on the CIPP model for the “3 Health for a CCA-Free Society” Manual (n = 56).

Topics	Mean	SD	Interpretation of opinion levels	Interpretation of consistency
1. The content of the social innovation manual for OV-CCA prevention and control				
The content of the innovation presentation is appropriate for all age groups.	5.00	0.00	Highest	Consistent
The language used in the innovation presentation is easy to understand.	4.78	0.42	Highest	Consistent
The font size and color are easy to read and appropriate.	4.65	0.48	Highest	Consistent
The design is colorful and eye-catching, with exciting illustrations.	4.81	0.38	Highest	Consistent
2. The inputs of the social innovation manual for OV-CCA prevention and control				
The accuracy of the content	5.00	0.00	Highest	Consistent
The coverage of content	5.00	0.00	Highest	Consistent
The appropriateness of the order of content presentation in the manual	4.73	0.45	Highest	Consistent
3. The process of social innovation for OV-CCA prevention and control				
The knowledge of lecturers/or speakers on transferring the innovation	5.00	0.00	Highest	Consistent
The duration of the innovation presentation	4.90	0.31	Highest	Consistent
The ability to transfer the innovation	4.82	0.38	Highest	Consistent
4. The outputs of the social innovation for OV-CCA prevention and control				
Being able to evaluate the knowledge and understanding of this social innovation before/after the implementation of the innovation	5.00	0.00	Highest	Consistent
Being able to disseminate/transfer knowledge	4.84	0.37	Highest	Consistent
Total mean of all items	4.88	0.26	Highest	

CCA, Cholangiocarcinoma; OV, *Opisthorchis viverrini;* SD, Standard deviation

## Author Contribution Statement

CT, OS, and NS conceived and designed the study. CT, GS, and NS coordinated and facilitated fieldwork in the community setting. CT, RJ, and NS collected the data. CT and NS carried out the analyses. CT and NS reviewed initial drafts of the manuscript. All authors contributed to the writing and revisions of the manuscript and approved the final version.
